# Elastic Fiber Programming for Simplified Pneumatic Control in Soft Robots

**DOI:** 10.1002/advs.202501477

**Published:** 2025-04-24

**Authors:** Xiaoli Yang, Tao Jin, Shiwei Tian, Jieyu Wang, Sicheng Yi, Yue Wang, Long Li, Yangqiao Lin

**Affiliations:** ^1^ Shanghai Key Laboratory of Intelligent Manufacturing and Robotics School of Mechatronic Engineering and Automation Shanghai University Shanghai 200444 China; ^2^ School of Electrical Engineering and Automation Anhui University Anhui 230601 China; ^3^ School of Future Technology Institute of Artificial Intelligence Shanghai University Shanghai 200444 China

**Keywords:** actuation control simplification, elastic fiber programming, modularization, pneumatic response characteristics, pneumatic soft robots

## Abstract

Conventional pneumatic soft robots require complex control systems with multiple valves and circuits due to coupled structural and pneumatic dynamics. A strategy is presented to program the pneumatic response characteristics of soft actuators by embedding elastic fibers with tailored materials and pre‐stretch ratios, enabling structurally identical modules to exhibit distinct pressure‐driven responses. A 6 mm‐diameter actuator prototype demonstrates this paradigm, achieving a 2632% load ratio and 225°/s bending speed at a material cost of 9 cents. Crucially, a single pneumatic input suffices for multi‐step actuation—shown in pipeline‐climbing robots, omnidirectional crawlers, and grippers adapting to objects from 10 to 450 g. This fusion of material programming and mechanical intelligence simplifies control architectures while enhancing functionality, offering a scalable path toward deployable soft robotics.

## Graphical abstract

This study introduces a novel approach to designing pneumatic soft actuators by leveraging elastic fiber programming to achieve tailored constitutive equations. Using pre‐stretched elastic fibers with varying properties and modurization, the soft robots can demonstrate diverse motions from a single pressure source. This simplifies control systems while enhancing versatility and functionality.

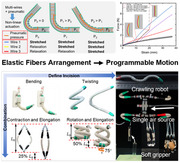

## 1 Introduction

Pneumatic actuation is widely employed in soft robotics due to its lightweight, high output force, and rapid response.^[^ ^1^, ^2^, ^3^, ^4^ ^]^ Due to their inherent compliance and continuous deformation capabilities, pneumatic soft robots can safely and adaptively interact with humans and unstructured environments, enabling them to accomplish tasks that are difficult for conventional rigid robots. Such tasks include grasping objects of various shapes and navigating confined spaces with agility.^[^ ^5^, ^6^, ^7^, ^8^, ^9^ ^]^

Pneumatic soft actuators primarily exhibit extension, bending, and torsion movements,^[^ ^10^ ^]^ with their actuation response governed by the actuator's structural configuration and the inherent constitutive mechanical properties of the material. Common structures for pneumatic soft actuators encompass fiber‐reinforced configurations,^[^ ^11^, ^12^, ^13^, ^14^, ^15^, ^16^, ^17^, ^18^ ^]^ elastic chamber designs,^[^ ^19^, ^20^, ^21^, ^22^, ^23^ ^]^ corrugated forms,^[^ ^24^, ^25^, ^26^, ^27^ ^]^ and foldable structures.^[^ ^28^, ^29^, ^30^, ^31^ ^]^ Currently, the fabrication of pneumatic soft actuators with different configurations typically requires separate molds for casting. This approach results in high production costs and time‐consuming fabrication processes. While 3D printing technology facilitates rapid manufacturing,^[^ ^32^ ^]^ the actuation response characteristics of pneumatic actuators remain fixed once their structural configuration is determined. Bishop‐Moser et al.^[^ ^11^ ^]^ proposed an intuitive selection chart for the design of fiber‐reinforced elastic actuators to enhance the efficiency of structural design. However, the inextensible fibers limit their applicability across diverse scenarios.

In practical applications, soft robots are often required to exhibit orderly and complex motion patterns. Currently, this is typically achieved by integrating multiple actuators.^[^ ^33^, ^34^, ^35^, ^36^ ^]^ However, each actuator requires an independent pneumatic control system, including solenoid valves, control circuits, and pumps, which leads to complex and redundant systems. Such complexity may constrain the soft robot's range of motion and flexibility.^[^ ^37^ ^]^ To address these challenges, Some researchers have designed programmable soft actuators by altering the structure or force of external constraints, thereby facilitating the realization of complex motion patterns.^[^ ^38^, ^39^, ^40^, ^41^, ^42^, ^43^ ^]^ This approach obviates the need for complex electronic components and preserves the actuators’ overall compliance by eliminating the incorporation of rigid elements. However, the fabrication process is time‐consuming and incurs significant costs. Some researchers have designed portable and embedded pneumatic valves based on flow control principles,^[^ ^44^, ^45^, ^46^, ^47^, ^48^, ^49^ ^]^ thereby simplifying the overall pneumatic control complexity and enhancing system reliability. However, the inherent rigidity of these embedded valves compromises the actuator's compliance. Furthermore, several studies have demonstrated that by manipulating the channel dimensions and elastic modulus differences,^[^ ^20^, ^37^ ^]^ soft actuators with varying pneumatic response characteristics can be designed, enabling orderly motion control under a single pneumatic input. However, the current strategies that leverage fluid viscosity and pre‐strain are relatively complex and lack straightforward design solutions for editing motion sequences.

Inspired by the flexion‐extension mechanism of spider legs, this work proposes an approach to simplify the control architecture of pneumatic soft actuators by tailoring their pneumatic response characteristics through elastic fiber programming. By strategically selecting elastic fibers of different types and adjusting their pre‐stretch ratios, the actuator's pressure response is precisely tuned. This enables a single pneumatic input to produce predictable, sequential, and complex motion patterns, effectively reducing the need for multiple control components. The pre‐integrated elastic fibers within the actuator make the structure more compact and enhance the output force and response speed of the actuator. Coupled with an elastic chamber structure, this approach facilitates a range of motion forms, including bending, torsion, extension, and combinations of these motions through specific actuator structures or combinations of multiple actuator modules. The compactness and programmability of these actuators enable their modular integration into various soft robots, such as pipeline crawling robots, omnidirectional crawling robots, and multifunctional grippers. These robots can perform diverse motion patterns with a single pneumatic input source, significantly simplifying the entire pneumatic control systems and expanding potential applications for pre‐deformed soft actuators.

## 2 Results

### 2.1 Design and Operation Principle of the Programmable Soft Actuator

**Figure 1:**
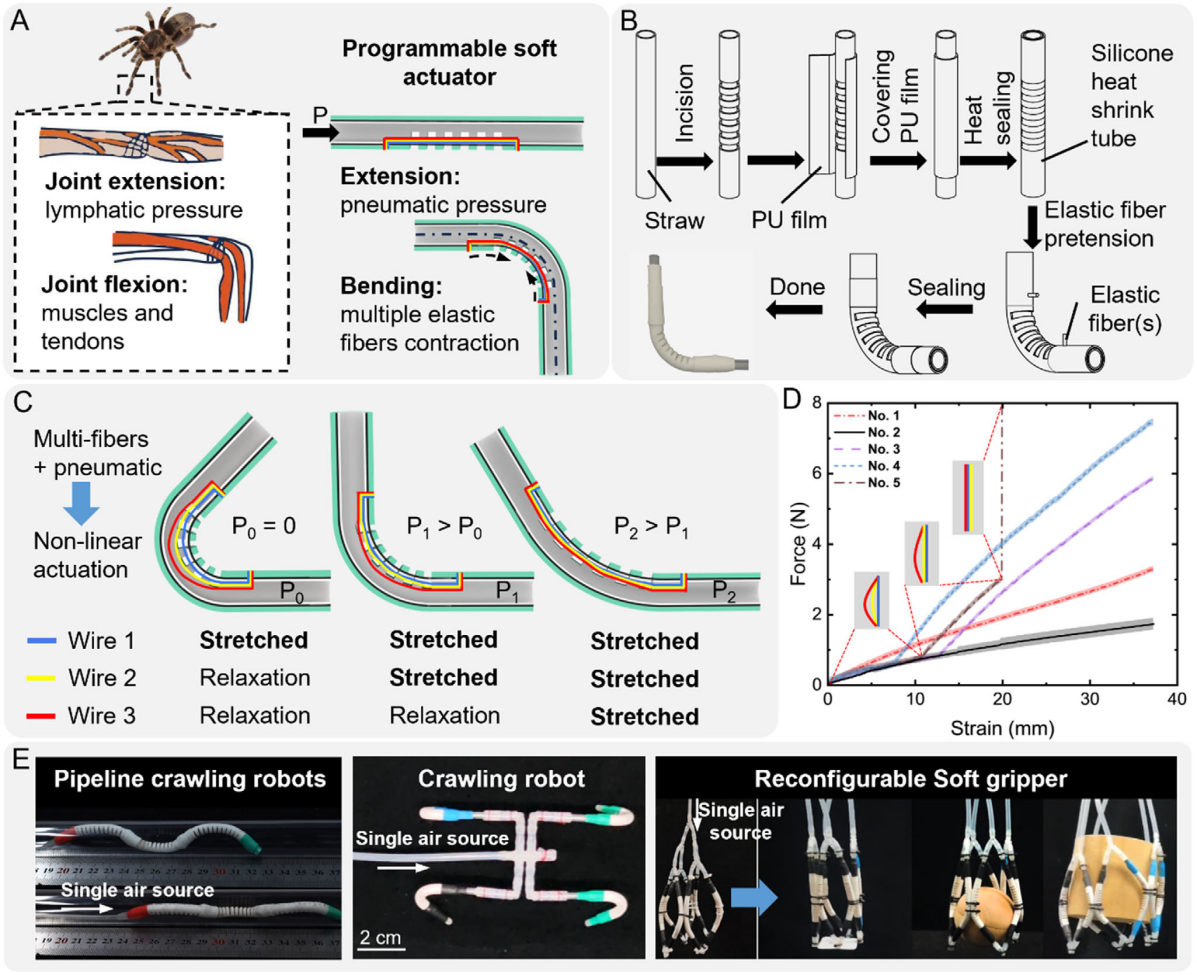
Bioinspired design and applications of the programmable soft actuators. A) The working principle of a soft actuator inspired by the extension‐flexion mechanism of a spider's leg. B) The materials and manufacturing process of the soft actuator. C) The programmable principle of the soft actuator. D) The “force‐strain” curves of six fiber groups with different materials and initial lengths. No. 1 to No. 6 represents 30 mm TPU fiber, 30 mm Latex fiber, 30 mm Latex and 43 mm TPU fibers, 30 mm Latex and 37 mm TPU fibers, 30 mm Latex and 41 mm TPU and 50 mm non‐elastic fibers, respectively. E) The demonstration of soft actuators' application scenarios.

The flexion‐extension mechanism of spiders' walking legs fundamentally relies on the interaction between the hydraulic pressure of hemolymph and the flexor muscles. The extension capability arises from a flexible joint located between rigid exoskeletal segments, which is equipped with a foldable ventral joint membrane. The spider pumps hemolymph into multiple channels within the exoskeleton, causing the joint membrane to inflate and unfold, which drives leg extension. Conversely, leg flexion is achieved through the contraction of numerous flexor muscles within the joint, as shown in **Figure** **1**A. Inspired by this biological mechanism, we designed a pneumatic‐elastic fibers hybrid actuator. The actuator features evenly distributed incisions along one side and incorporates three pre‐stretched elastic fibers on the same side internally. When actuated by pneumatic pressure, the internal pressure increases causing the actuator to extend. Without pneumatic input, the contraction force exerted by the pre‐stretched elastic fibers on one side induces the actuator to bend.

The components of the soft actuator include straws, PU (polyurethane) film, silicone heat‐shrink tubing, and elastic fibers, all of which are readily available and low‐cost (with an average cost per actuator of 9 cents). The fabrication process is illustrated in Figure 1B and Figure S1 (Supporting Information). A laser cutter was used to create uniform incisions on the surface of the straw and the silicone heat‐shrink tubing. The incisions on the straw were then sealed with adhesive PU film. Subsequently, the silicone heat shrink tubing was placed over the straw, and a heat gun was used to ensure tight adhesion to the straw, enhancing the actuator's maximum pressure resistance. To maintain consistent bending performance, the incisions on the silicone heat‐shrink tubing should be aligned with those on the straw. Small holes were created at both ends of the processed straw using a soldering iron and pre‐stretched elastic fibers are secured to these holes. Finally, silicone adhesive was applied to seal the holes of the soft actuator. To fabricate actuators with distinct response characteristics, it is only necessary to change the type of elastic fibers and their pre‐stretching rates.

Inspired by the flexion‐extension mechanism of a spider's walking legs, this study introduces a motion‐programmable strategy for actuators: elastic fibers of varying materials and diameters exhibit distinct linear “force‐strain” response characteristics. A tailored nonlinear “force‐strain” response curve can be constructed by combining multiple types of elastic fibers with different pre‐stretching rates, endowing the actuator with specific pressure response characteristics. As shown in Figure 1C, wire 1 and wire 2 are elastic fibers, while wire 3 is non‐elastic. In the initial state, wire 1 is pre‐stretched, while wire 2 and wire 3 remain relaxed, and the internal pressure of the actuator *P*_0_ = 0. At this stage, the actuator remains bent, driven exclusively by the contraction force of wire 1. When the internal pressure of the soft actuator increases to *P*_1_ (*P*_1_ > *P*_0_), the actuator gradually extends, overcoming the tensile force of wire 1. As the extension angle increases, wire 1 and wire 2 become taut, jointly contributing to the tensile force. With further increases in input pressure to *P*_2_ (*P*_2_ > *P*_1_), the extension angle increases further, and all three wires—wire 1, wire 2, and wire 3—become tensioned. Since wire 3 is non‐elastic, it limits further extension, and the actuator's bending angle stabilizes. By adjusting the pre‐tension of elastic fibers made of different materials (e.g., TPU, latex) to various degrees, specific “force‐strain” characteristics can be formulated, enabling actuators to exhibit tailored pressure response characteristics. Examples of combined “force‐strain” curves are illustrated in Figure 1D (see Figure S2, Supporting Information).

In this study, soft actuators with varying pressure response characteristics were achieved through different arrangements of elastic fibers. By combining these actuators, a wide range of motion forms can be achieved, enabling applications such as pipeline crawling, omnidirectional locomotion, operations in confined spaces, and enveloping grasping, as depicted in Figure 1E.

### 2.2 Parametric Design and Optimization of the Programmable Soft Actuator

**Figure 2:**
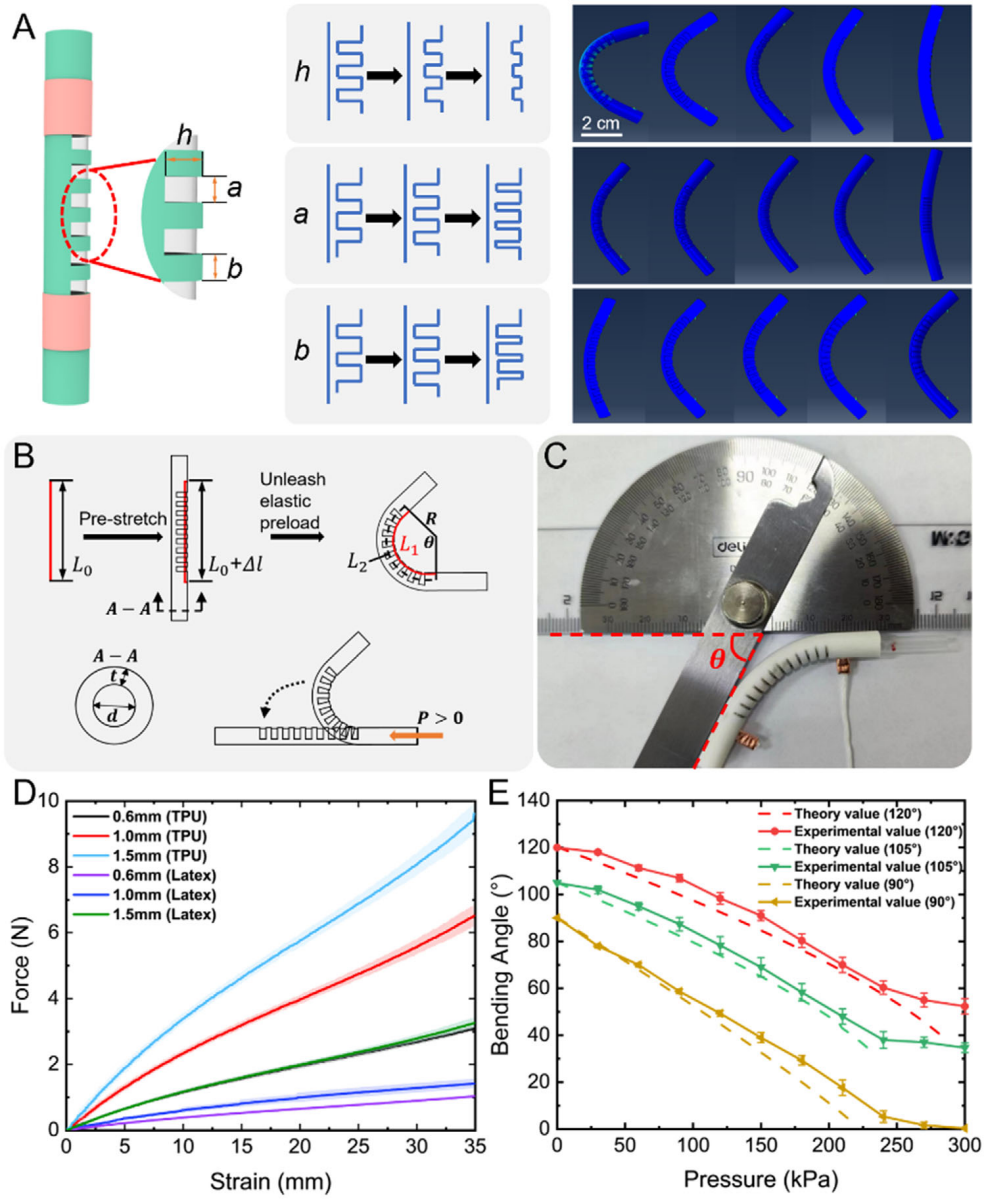
Structural optimization and analysis of pneumatic response characteristics of soft actuators. A) The influence of different cut depths, widths, and spacings on bending performance. B,C) The geometric relationship between the initial bending angle of the soft actuator and the pre‐stretch ratio of the elastic fiber. D) The force‐strain curves of different elastic fibers. E) The theoretical and experimental response characteristics of the soft actuators with initial bending angles of 90°, 105°, and 120° (corresponding to different pre‐stretch ratios) under pneumatic pressure ranging from 0 to 300 kPa.

The actuation response characteristics of soft actuators are influenced by the structural configuration and constraints imposed by elastic fibers. To maximize the bending angle variation of the soft actuator under pneumatic actuation, we first optimized its structural design, including the incision depth *h*, incision width *a*, and incision spacing *b*, as shown in Figure 2A. The initial bending angle of the actuator is determined by its intrinsic structure and the contraction force induced by the pre‐stretching of the elastic fibers. A greater initial bending angle results in a larger bending angle variation under pneumatic actuation. To efficiently evaluate the influence of these design parameters on the initial bending angle of the soft actuator, ABAQUS simulations were utilized to model actuator bending angles under a constant pre‐tension force of *F_pre_* =  3 *N*, thereby enabling the rapid optimization of design parameters to inform actuator development. The simulation resolutions are presented in Figure 2A. Incision depth *h* was varied as 0.2, 1.2, 2.2, 3.2, and 4.2 mm. The results indicate that an increase in incision depth leads to a larger bending angle under a constant pre‐tension force. However, excessive depth may damage the straw or silicone tube during actuation. Experimental results identified an optimal incision depth of 3.2 mm. Incision width *a* was set at 0.5, 1.0, 1.5, 2.0, and 2.5 mm. Simulations showed that when the incision width was 0.5 mm, the initial bending angle was only 10°. For widths exceeding 1.0 mm, the bending angle exhibited minimal variation. Considering manufacturing precision and actuator sealing, the ideal incision width was determined to be 1.5 mm. For incision spacing *b*, values of 0.5, 0.75, 1.0, 1.25, and 1.5 mm were analyzed. The results demonstrated minimal variation in initial bending angle for spacing between 0.5 and 1.25 mm. Beyond 1.25 mm, the initial deformation angle decreased due to increased material resilience. In experiments, actuators with excessively narrow incision spacing experienced rupture at the incision position under pneumatic pressure. Therefore, 1.25 mm was selected as the optimal incision spacing.

Once the actuator's structural design parameters are determined, the initial bending angle is significantly influenced by the pre‐stretch ratio of the elastic fiber. As shown in Figure 2B, an elastic fiber with an initial length *L*_0_ is pre‐stretched to *L*_0_ + Δ*l* and fixed at both ends of the actuator. The stored elastic potential energy causes the fiber to contract, leading to initial bending deformation of the actuator, resulting in a new fiber length *L*_1_, a bending angle θ, and a curvature radius *R*. The initial bending angle of the actuator can be directly used to characterize the pre‐stretch ratio of the elastic fibers through geometric relationships, as illustrated in Figure 2C.

To determine the response characteristics of the actuator under pneumatic pressure, it is essential to obtain the constitutive relationships of elastic fibers with varying materials and diameters, specifically their “force‐strain” response characteristics. We analyzed the axial tensile “force‐strain” curves of three types of TPU and latex fibers, as shown in Figure 2D. The experimental data reveal that elastic fibers of different diameters and materials exhibit distinct elastic coefficients, indicating that selecting various types of elastic fibers facilitates the design of soft actuators with tailored response characteristics. We conducted tests to measure the bending angle variations of these actuators with initial bending angles of 90°, 105°, and 120° (corresponding to different pre‐stretch ratios for TPU fiber with a diameter of 1 mm) under pneumatic pressure ranging from 0 to 300 kPa, as depicted in Figure 2E. The experimental results demonstrate that soft actuators with different initial bending angles exhibit distinct pneumatic response characteristics. This indicates that the pneumatic response characteristics of the soft actuators can be defined by designing the pre‐stretch ratio of the elastic fibers. When the driving pressure reaches 300 kPa, the elastic fibers inside the soft actuator remain at maximum elongation. At this point, the elastic force generates a significant torque on the actuator. Due to the actuator's relatively low stiffness, the input pressure cannot fully counteract this torque, resulting in a reduced change in the bending angle.

During the bending deformation process induced by pneumatic actuation, energy conservation involves the elastic potential energy generated by the actuator's bending deformation, the elastic potential energy from the stretched elastic fiber, and the work performed by the pneumatic pressure. We used the energy method to theoretically analyze the relationship between pneumatic pressure *P* and the bending angle θ. Initially, the elastic potential energy *U_L_* generated by the stretched elastic fiber balances with the elastic potential energy *U_R_* of the actuator's bending. Subsequently, the actuator changes the bending angle under the applied input pressure *P* until it reaches a new equilibrium state. During this process, the elastic potential energies *U_L_* and *U_R_* also exhibit corresponding changes. Given the significant deformation characteristics of the actuator, the Yeoh strain energy density model was applied to describe its deformation behavior, resulting in the following strain energy density function:

(1) $$W=C_{10}\left(I_{1}-3\right)+C_{20}{\left(I_{1}-3\right)}^{2}$$

Here, *C*_10_ and *C*_20_ are parameters of the Yeoh model which can be obtained by stree‐strain experiment (see Figure S3, Supporting Information), and *I*_1_ represents the first invariant of the Cauchy‐Green strain tensor.

Without any external forces, the system reaches a stable state once the inflation process of the pneumatic soft actuator's cavity is complete. Using the principle of virtual work, the following equation is formulated:

(2) $$P_{i}dV_{c}+V_{r}dW=\sum_{i=1}^{n}k_{i}x_{i}dx_{i}$$

where *P_i_* represents the input pressure, *V_c_* represents the cavity volume when the actuator's bending angle is θ, *V_r_* represents the volume of the actuator material, *k_i_* represents the elastic coefficient of the *i*‐th elastic fiber, and *x_i_* denotes the deformation of the *i*‐th elastic fiber.

From Equation (2), the relationship between the actuator angle θ and the input pressure *P_i_* can be expressed as follows:

(3) $$P_{i}=\frac{8\sum_{i=1}^{n}k_{i}\left(d+t\right)x_{i}-8\pi \left(dt+t^{2}\right)\left[L_{0}+\Delta l-\left(\frac{d}{2}+t\right)\theta \right]\frac{dw}{d\theta }}{\pi d^{2}\left(d+2t\right)}$$

where *t* represents the wall thickness of the soft actuator. Therefore, by defining the elastic fiber material and pre‐stretch length, the initial bending angle and pneumatic response characteristics of the actuator can be predicted. Inspired by Wang, et al,^[^ ^18^ ^]^ this work has extended the range of elastic fiber materials and pre‐stretch lengths and developed a theoretical framework to predict the deformation, thereby expanding the design space and facilitating deformation design for various applications. Further details are available in Figure S4, Supporting Information.

The theoretical bending angle variations of the soft actuators with initial bending angles of 90°, 105°, and 120° as the input pressure increases from 0 to 300 kPa are illustrated in Figure 2E. The mathematical model effectively predicts the pressure‐angle relationship for the soft actuators across different initial bending angles. When the pressure exceeds 240 kPa, the increased tension in the elastic lines enhances the frictional force between the elastic fibers and the actuator's inner wall. Consequently, additional work is required to overcome this friction.

### 2.3 Programmable Design of the Soft Actuator

**Figure 3:**
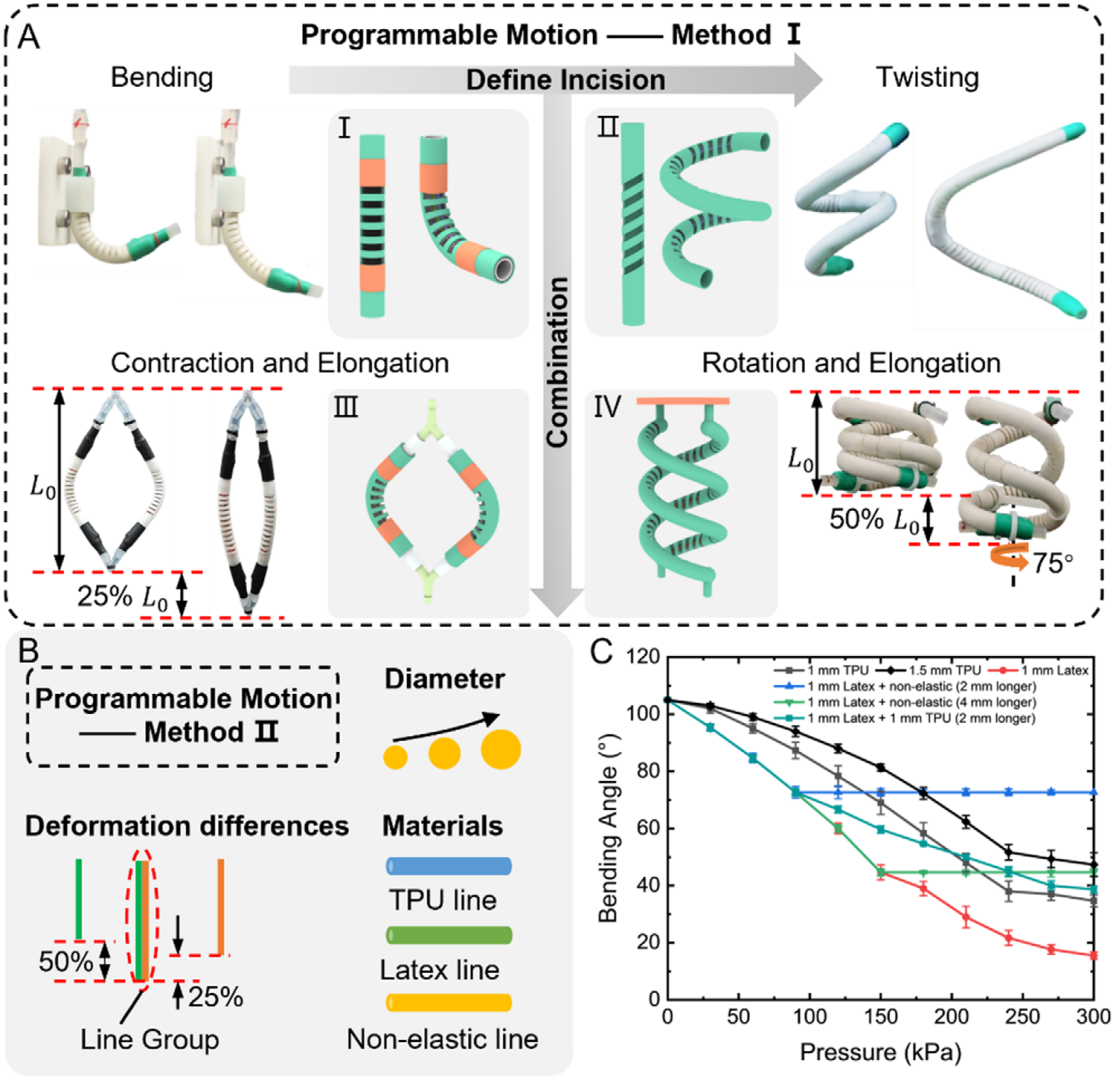
Programmable design of soft actuators. A) Programmable motion of the soft actuator, enabled by designing specific incision patterns or their combinations. B) Programmable motion of the soft actuator, achieved by integrating elastic fibers with varying diameters, pre‐stretch ratios, and materials. C) The pneumatic response characteristics of soft actuators with an initial bending angle of 105° while embedded with different elastic fiber sets. Each actuator was embedded with elastic fibers of varying properties or pre‐stretch ratios, under driving pressures ranging from 0 to 300 kPa. Data from three tests per actuator was used to calculate the means and standard deviations.

The motion programming of soft actuators can be achieved by modifying the incision patterns of soft actuators or combining multiple soft actuators, as shown in Figure 3A and Video S1 (Supporting Information). The degree of the incision relative to the longitudinal axis of the tube determines the type of motion: a perpendicular incision results in bending deformation, whereas a non‐perpendicular incision induces twisting motion, as illustrated in Figure 3A (I, II). Additionally, combining multiple actuators facilitates the transition from simple to complex motion forms. For instance, combining two actuators shown in Figure 3A (I) allows for both lateral contraction and longitudinal elongation, as shown in Figure 3A (III). Similarly, combining two actuators from Figure 3A (II) permits rotation and elongation, as depicted in Figure 3A (IV).

Programmable motion in soft actuators can also be achieved by tailoring their pneumatic response characteristics through elastic fiber design. As shown in Figure 3B, by utilizing single or multiple elastic fibers with varying materials, diameters, or pre‐stretch ratios, specific nonlinear “force‐strain” response relationships can be achieved. This enables the construction of soft actuators with distinct pneumatic response characteristics. We designed six soft actuators, each with an initial bending angle of 105°, but differing in the quantity, diameter, material, or pre‐stretch ratio of the internal elastic fibers. The variations in bending angles under pneumatic pressures ranging from 0 to 300 kPa are shown in Figure 3C. Each actuator exhibits distinct pneumatic response characteristics, and when subjected to pneumatic actuation from 0 to 300 kPa, the coefficient of variation of the bending angle across all actuators remains below 5.7%, indicating consistent deformation control accuracy. Due to the lower elastic modulus of latex fibers, actuators using latex fibers exhibit greater bending angle changes with variations in pressure. Actuators with different fiber combinations display unique pressure response characteristics, and adjusting the pre‐tension in fibers within the same configuration yields different pressure responses.

### 2.4 Dynamic and Output Performance

**Figure 4:**
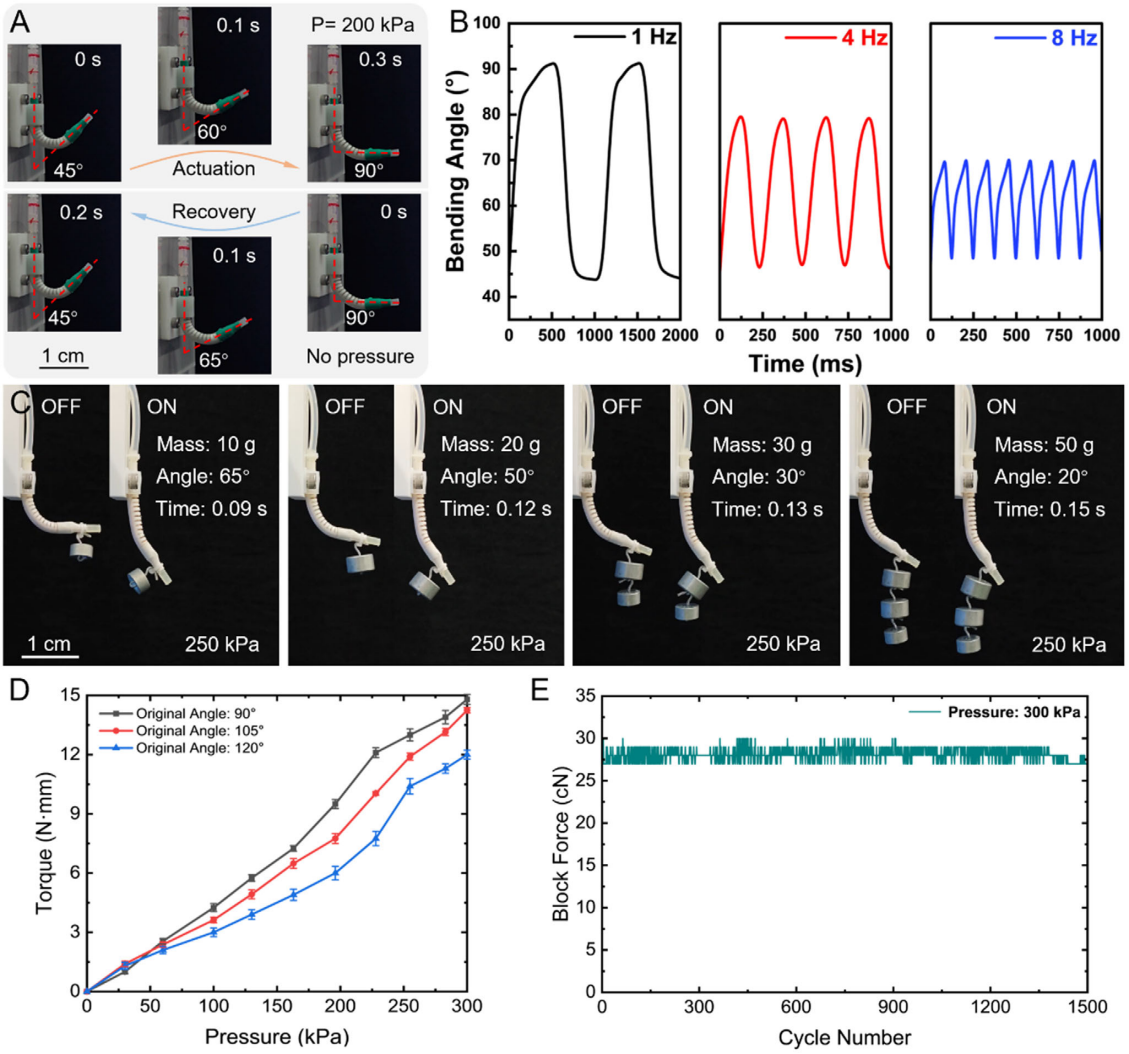
Performance evaluation of response speed and output force of the soft actuator. A) The actuation response speed and recovery speed of the soft actuator. The soft actuator initially bent at the angle of 45° and transitioned to 90° within 0.3 s when subjected to an input pressure of 200 kPa. With no input pressure, the actuator returns to its initial state within 0.2 s. B) Response speed of the soft actuator under different actuation frequencies. The change in bending angle indicates that as the actuation frequency increases from 1 to 8 Hz, the bending angle variation decreases from 45° to 22°. C) The load capacity test of the soft actuator. Bending angle variation and response time of the soft actuator under external loads ranging from 10 to 50 g when subjected to an input pressure of 250 kPa. D) Output torque under different applied pressures (initial bending angles of the actuators: 90°, 105°, and 120°). E) Reversible cyclic changes of the blocking force over 1500 cycles of the actuator under 300 kPa actuation pressure.

The dynamic response speed of soft actuators constitutes critical performance indicators. As depicted in Figure 4A, at an input pressure of 200 kPa, an actuator initially bent at 45° deforms to a balanced state at 90° within 0.3 s, yielding a response rate of 150° s^−1^. The actuator reverted to its initial bending state within 0.2 s, producing a recovery rate of 225° s^−1^. Figure 4B demonstrates the time‐dependent angle variations of the actuator at driving frequencies between 1 and 8 Hz (see Figure S5 and Video S2, Supporting Information). As the driving frequency increases, the amplitude of bending angle variation gradually decreases, where the peak angles do not attain the maximum of the initial state, and the troughs do not descend to the minimum of the static response. This occurs due to insufficient time for complete inflation and deflation cycles. At lower frequencies, the actuator possesses sufficient time for full inflation and deflation, enabling it to reach its maximum deformation angle in each actuation cycle.

The output force of soft actuators is also a significant performance metric. As shown in Figure 4C, weights ranging from 10 to 50 g were affixed to the end of the soft actuator, and its bending angle variation was recorded under an input pressure of 250 kPa for different load conditions. As the load at the actuator's end increased, the range of bending angle change gradually decreased. When the mass was 50 g, the bending angle variation decreased from ≈65° (with a 10 g load) to ≈20° (see Video S3, Supporting Information). The output torque of actuators with different initial bending angles also varied according to input pressure. Tests were conducted on actuators with initial bending angles of 90°, 105°, and 120°, under input pressures ranging from 0 to 300 kPa, with the results shown in Figure 4D and Figure S6 (Supporting Information). The actuator with an initial bending angle of 90° exhibited the highest output torque of 14.8 N·mm. Increased initial bending angles imply greater pre‐tension, subsequently requiring more input pressure to balance, thereby causing a gradual decrease in output torque. Additionally, through stability tests conducted over 1500 actuation cycles, as shown in Figure 4E, it was demonstrated that the soft actuator exhibits good repeatability and durability, which indicates its robust performance and suitability for practical applications.

### 2.5 Applications

Soft actuators with different elastic fiber arrangements demonstrate distinct constitutive behaviors, leading to different pneumatic response characteristics under the same input pressure. By segmenting a single soft actuator into multiple sections, each with a distinct elastic fiber configuration, or by combining multiple actuators with different fiber arrangements, diverse motion forms can be realized using a single pneumatic input source.

#### 2.5.1 Pipeline Crawling Soft Robot

**Figure 5:**
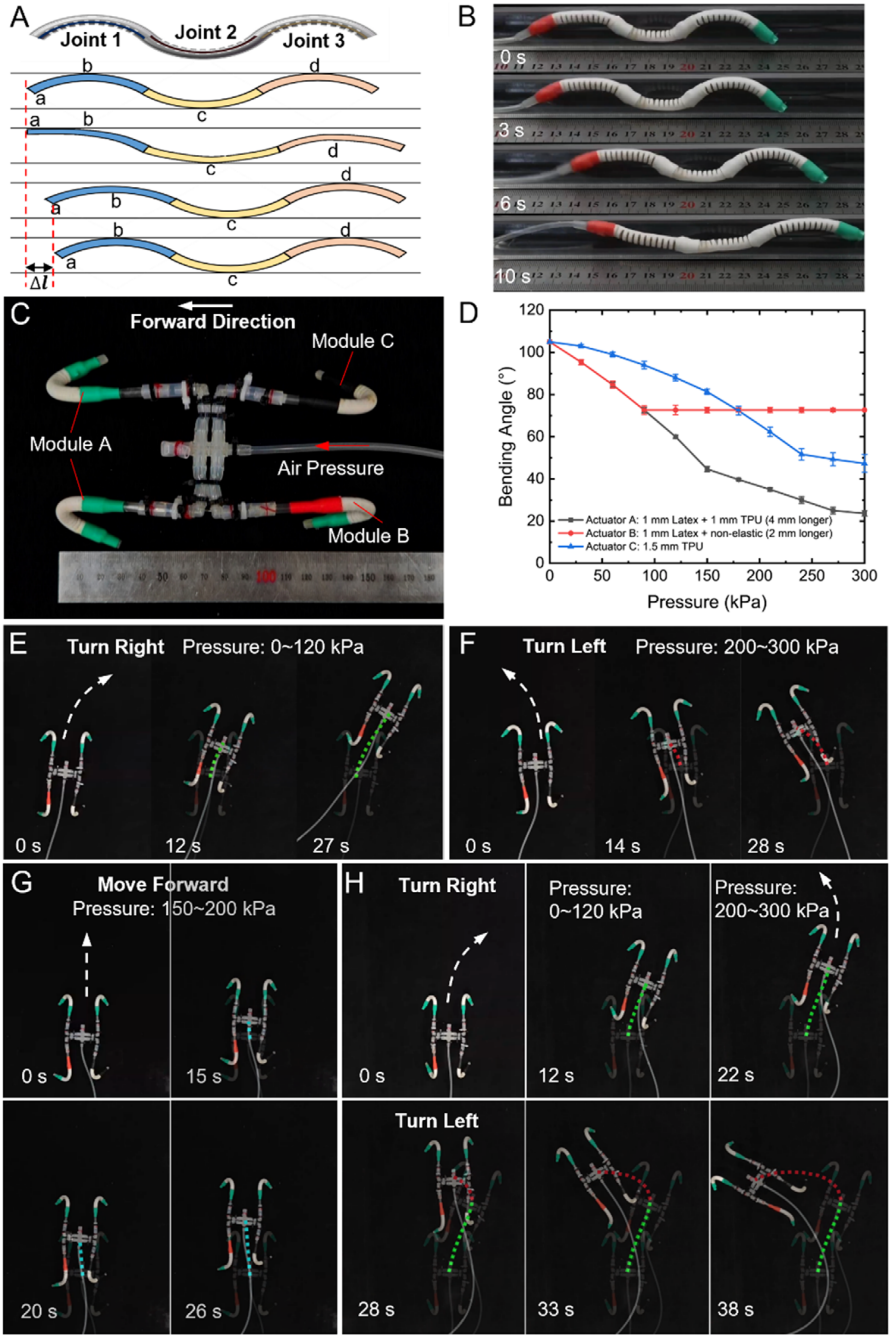
Soft crawling robots are composed of actuators with different elastic fiber arrangements or elastic fiber groups. A) Operational principle of a single cycle for the pipeline crawling soft robot. This robot consists of a single actuator, which is divided into three sections, each embedded with a pre‐stretched elastic fiber of different stiffness coefficients. B) The soft robot crawling inside a transparent acrylic pipe with a diameter of 15 mm, driven by a single pneumatic input of 200 kPa and a frequency of 4 Hz. C) Omnidirectional crawling soft robot driven by a single pneumatic source, composed of three soft actuator modules with distinct pneumatic response characteristics. D) Pneumatic response characteristics of the three soft actuator modules and their elastic fiber arrangements. E–G) The soft robot achieves right turn, left turn, and straight‐line crawling under different input pressures (0–120 kPa, 200–300 kPa, and 150–200 kPa) at a driving frequency of 1 Hz. H) The soft robot performing continuous “S‐shaped” crawling.

Figure 5A illustrates the working principle of the pipeline crawling robot driven by a single pneumatic source. This soft robot consists of a single actuator divided into three segments, each functioning as a joint. Each joint is equipped with elastic fibers of stiffness coefficients *k*_1_, *k*_2_, and *k*_3_ (*k*_1_ < *k*_2_ < *k*_3_), and ensuring uniform initial bending angle across the joints. Under a single pneumatic input, the three joints exhibit different bending deformation characteristics at the same input pressure, resulting in differential deformation that allows the robot to crawl within an acrylic pipeline with a diameter of 15 mm. In one cycle, as shown in Figure 5A, the pipeline crawling robot contacts the pipeline wall at points b, c, and d when not actuated. When pressure is applied, joints 1, 2, and 3 simultaneously extend. Due to the differences in the elastic fibers, the bending radii satisfy *r*_1_ > *r*_2_ > *r*_3_, causing point d to lift off the wall while points a and b make contact, with joint 1 exhibiting the largest extension angle. When the input pressure returns to zero, joint 3 retracts faster than joint 1, causing the robot to shift right by a distance Δ*L* under the effect of friction. With periodic pneumatic input, the robot achieves a crawling speed of 3.5 mm s^−1^, as depicted in Figure 5B and Video S4 (Supporting Information). By adjusting the initial bending angle of joints, the robot can adapt to pipelines of varying diameters, rendering it suitable for applications such as pipeline inspection and maintenance.

#### 2.5.2 Omnidirectional Crawling Robot

To expand the applications of programmable soft actuators, we introduced the concept of modularity, designing an omnidirectional crawling soft robot, as shown in Figure 5C. The crawling robot can perform left‐turn, right‐turn, and forward motion through a single pneumatic input. Its movement is primarily governed by the three distinct soft actuator modules A, B, and C, which have the same initial bending angle but different pneumatic response characteristics. Module A is symmetrically positioned at the front of the actuator, ensuring consistent forward movement during operation. Modules B and C coordinate to control the robot's forward motion, left turns, and right turns. When the bending angle variation of Module B is greater than that of Module C, the combined motion of actuators B and C results in the soft robot turning right. When the bending angle variation of Module B is smaller than that of Module C, the combined motion of actuators B and C results in the soft robot turning left, as shown in Figure S7 (Supporting Information). When the bending angle variations of Module B and Module C are identical, the combined motion of actuators B and C allows the soft robot to move straight forward. The bending angle versus input pressure response characteristics for these three soft actuators are illustrated in Figure 5D. Within the input pressure range of 0–300 kPa, the bending angle variation of Module A remains the largest and is symmetrically distributed at the front of the soft actuator, ensuring that the soft robot consistently moves forward during operation. Within the input pressure range of 0–120 kPa, the bending angle variation of actuator B is greater than that of actuator C, resulting in the soft robot turning right, as shown in Figure 5E. Within the input pressure range of 200–300 kPa, the bending angle variation of actuator B is smaller than that of actuator C, resulting in the soft robot turning left, as shown in Figure 5F. Within the input pressure range of 150–200 kPa, the bending angle variations of actuator B and actuator C are similar, resulting in the soft robot moving straight forward, as shown in Figure 5G. Additionally, we performed a continuous “S‐shaped” crawling experiment with the soft robot, during which the input pressure range was maintained at 0–120 kPa with a frequency of 1 Hz from 0 to 38 s; in this phase, the soft robot crawled to the right. Then the pressure regulator was adjusted to set the pressure range to 200–300 kPa, enabling the soft robot to change direction and crawl to the left during this phase, as shown in Figure 5H (see Video S5, Supporting Information). The experimental results show that the left turn radius of the soft robot is smaller than the right turn radius. This is attributed to the more significant angular variation between Module B and C during left turns compared to right turns.

By leveraging the differences in the pneumatic response characteristics of the actuator modules, the motion programming of a crawling robot can be achieved by simply adjusting the output pressure of a single air source. This approach significantly simplifies the pneumatic control system. The input pressure can be modified, or the modules can be substituted to suit specific task requirements or environmental conditions, enabling diverse movement modes, and enhancing the robot's functional diversity and application range. By integrating various actuator modules, the crawling robot can exhibit enhanced efficiency and adaptability when executing complex tasks.

#### 2.5.3 Multi‐Functional Flexible Gripper

A soft gripper is a commonly utilized robotic end‐effector, known for its significant flexibility and adaptability, allowing it to function effectively in unstructured environments. Since the actuator itself functions as a pipeline, it is easily modularized and assembled through the use of pipe joints. Utilizing reconfigurable combinations, we transformed the soft pneumatic actuator (SPA) into different kinds of soft robotic grippers capable of managing diverse objects and tasks (see Video S6, Supporting Information).

**Figure 6:**
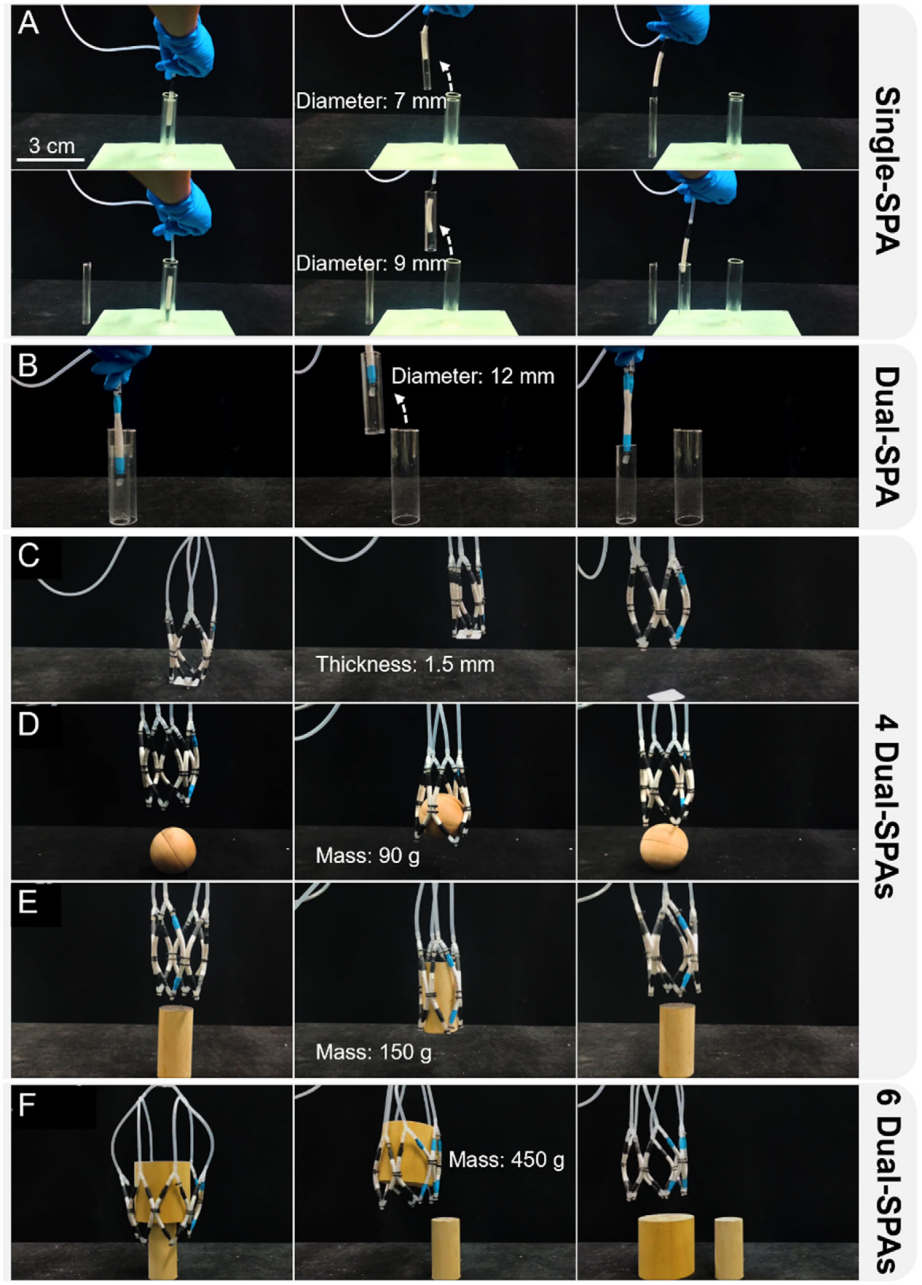
Soft robotic gripper designed for diverse applications through the reconfigurable combination of multiple soft actuators. A) A single soft actuator enabling zero‐power manipulation of tubular objects with a minimum inner diameter of 7 mm. B) A dual‐SPA formed by two soft actuators in parallel, achieving zero‐power grasping of tubular objects with a larger inner diameter of 12 mm. C–E) Soft gripper formed by four parallel dual‐SPAs, successfully grasping a 20 mm × 20 mm thin sheet with a thickness of only 1.5 mm, a solid wooden ball weighing 90 g, and a cylindrical wooden object weighing 150 g. F) A soft gripper formed by six parallel dual‐SPAs, capable of grasping a cylindrical wooden object with a diameter of 120 mm and weighing 450 g.

The soft actuator introduced in this study features a compact design, rapid response, and a diameter of only 6 mm, enabling it to function in confined spaces, such as grasping small tubular components within pipelines. As shown in Figure 6A, the soft actuator was initially pressurized to straighten and enter the cavity of the tubular component. Upon releasing the pressure, the actuator reverted to its original deformed state and made contact with the inner wall of the tubular component, thereby facilitating zero‐power manipulation. As the size of the tubular component increases, the number of actuators can be modified to accommodate changes in component dimensions, as depicted in Figure 6B.

A mesh‐based flexible gripper is designed using a multi‐actuator configuration strategy. The gripper consists of *n* dual‐SPAs which is shown in Figure 6B, with individual dual‐SPA arranged sequentially to create a grid‐like framework. This arrangement enables greater flexibility and more uniform force distribution, thereby enhancing grasping efficiency and adaptability. Figure 6C illustrates the multi‐SPA mesh gripper with *n*  = 4, which successfully grasped a thin component with dimensions of 20 mm × 20 mm and a thickness of only 1.5 mm. Additionally, the gripper demonstrated its ability to grasp both a spherical object (diameter: 75 mm, weight: 90 g) and a cylindrical object (diameter: 50 mm, height: 100 mm, weight: 150 g), as depicted in Figures 6D,E. With an actuator group of *n*  = 6, the mesh soft gripper could grasp a cylindrical object with a diameter of 120 mm, height of 100 mm, and weight of 450 g, achieving a load‐to‐weight ratio of 2000%. According to the size and weight of the object to be grasped, the actuator count *n* can be adjusted to improve the gripper's output force and gripping range.

## 3 Conclusion

This study introduces an innovative strategy for pneumatic soft actuators by leveraging elastic fiber programming to achieve tailored pneumatic responses under a single input. The design of soft robots often relies on structural morphology to achieve various motion patterns, with each part requiring an independent control system. Unlike conventional designs that rely on multiple pneumatic sources^[^ ^33^, ^34^, ^35^, ^36^ ^]^ or channel configurations,^[^ ^20^, ^37^ ^]^ this method simplifies the control architecture while enabling diverse motion patterns. Inspired by the flexion‐extension mechanism of spiders' walking legs, the proposed elastic fiber programming strategy utilizes fibers with different materials and pre‐stretch ratios to define the actuator's pneumatic response, thereby resolving the trade‐off between system integration and motion complexity without requiring extensive control circuitry.

Soft actuators with designed incisions achieve bending and twisting motions while combining multiple actuators enables complex motion forms such as twisting and elongation. Theoretical and experimental results confirm that elastic fiber integration allows precise customization of pneumatic response characteristics, expanding applications that require adaptability and precision. The proposed cost‐effective actuators maintain stable performance over 1500 actuation cycles. Their lightweight (1.9 g), compact (6 mm diameter) design, combined with programmable pneumatic responses, enhances their applicability. A performance comparison (Table 1) demonstrates the actuator's competitiveness in high‐performance soft robotics and simplified pneumatic control. The programmable and high‐performance characteristics of the soft actuator enable integration into various soft robots, including sequence‐driven pipeline‐climbing robots, omnidirectional crawling robots, and reconfigurable multi‐scale soft grippers. This approach simplifies the control architecture of pneumatic soft robots, providing a scalable solution for scenarios requiring high integration and low control complexity, such as minimally invasive surgical instruments and disaster rescue robots.

**Table 1:** Comparison of our proposed actuator with pneumatic programmable actuators in the literature.

References	Volume [cm^3^]	Mass [g]	Bend	Elongation	Twist	Load‐to‐weight Ratio	Respond Speed [°/s]	Programmable Principle	Sequential Motion Drive Mode
[17]	47.53	/	22.5	√	√	/	/	inflated structure	multi‐inputs
[19]	9.69	/	180	/	/	/	64	stiffness	single input
[32]	40.19	18.75	70	/	/	6.35	700	inflated structure	multi‐inputs
[36]	20.70	/	350	√	√	/	/	viscous flow	single input
[37]	5.70	/	120	√	√	/	11	inflated structure	/
[38]	3.80	/	216	√	√	16	1100	inflated structure	single input
[39]	86.48	80	300	/	/	/	/	inflated structure	/
[40]	0.32	18.9	360	√	√	/	/	inflated structure	multi‐inputs
[41]	12.06	/	180	/	/	/	/	stiffness	/
[45]	2.57	/	180	/	/	/	72	viscous flow	single input
this work	1.41	1.9	120	√	√	26.32	225	elastic fiber arrangement	single input

The simplicity of the proposed pneumatic soft actuator design allows for potential functional enhancements. The customization of pneumatic response characteristics currently relies on the arrangement of elastic fibers. Future advancements could integrate intelligent elastic materials whose mechanical properties can be altered via external stimuli such as electricity, heat, or light. This would enable real‐time reprogramming of the pneumatic response characteristics, further simplifying soft robot design and reducing the complexity of control systems.

## Conflict of Interest

The authors declare no conflict of interest.

## Supporting information



Supporting Information

Supplemental Video 1

Supplemental Video 2

Supplemental Video 3

Supplemental Video 4

Supplemental Video 5

Supplemental Video 6

## Data Availability

The data that support the findings of this study are available in the supplementary material of this article.
