# Supplementary material for: Elastic Fiber Programming for Simplified Pneumatic Control in Soft Robots
Source: Adv Sci (Weinh). 2025 Apr 24;12(25):2501477. doi: 10.1002/advs.202501477 (PMC12224970; doi:10.1002/advs.202501477)
Supplement: Supplementary file 1 — Supporting Information [file ADVS-12-2501477-s006.docx]

Supporting Information

**Elastic Fiber Programming for Simplified Pneumatic Control in Soft Robots**

Xiaoli Yang, Tao Jin, Shiwei Tian, Jieyu Wang, Sicheng Yi, Yue Wang, Long Li, and Yangqiao Lin*

**Figure S1. Materials and Fabrication Process of the Soft Actuator.**

**Figure S2. "Force-Strain" Test Experiments for Different Elastic Fiber Sets.**

**Figure S3. Mechanical Properties Testing of Silicone Tubing.**

**Theoretical model of pneumatic response characteristics of programmable soft actuator**

**Figure S4. Theoretical Predicted Effects of Elastic Fiber Arrangement and Input Pressure on Bending Soft Actuators.**

**Figure S5. Design of the Pneumatic Actuation Control Experimental Platform.**

**Figure S6. Soft Actuator Output Torque Testing Experiment.**

**Figure S7. Variations in the bending angles of Modules B and C under different pressure ranges.**

**Other Supplementary Material for this manuscript includes the following:**

**Video S1 (.mp4 format). Programmable design of the soft actuator.**

**Video S2 (.mp4 format). Actuation response speed and recovery speed of the soft actuator.**

**Video S3 (.mp4 format). The load-carrying capacity test of the soft actuator.**

**Video S4 (.mp4 format). A pipeline crawling robot driven by a single air source input.**

**Video S5 (.mp4 format). Omnidirectional crawling robot actuated by a single air source input.**

**Video S6 (.mp4 format). Reconfigurable soft robotic gripper designed for diverse applications.**

The materials used for the soft actuator include medical PU film (Jiangsu Hongsheng Bioengineering Co., Ltd.), silicone heat-shrink tubing (Zhongshan Jixin Electronic Materials Co., Ltd.), straws (Yiwu Shuangtong Daily Necessities Co., Ltd.), TPU fibers (Suqian Jianhui Accessories Co., Ltd.), and latex fibers (Jiangsu Great Textiles Co., Ltd.), as shown in Figure S2(A). The average cost of materials for each component and the total cost of a single soft actuator are presented in Figure S2(B). The fabrication process of the soft actuator is illustrated in Figure S2(C). First, the laser cutter (CW-3500, Shenzhen Shenming Daxin Laser Intelligent Equipment Co., Ltd.) is configured with appropriate cutting speed and power to make defined incisions on the straw and silicone heat-shrink tube. To prevent the laser from cutting through the materials, copper rods are inserted into the straw and heat-shrink tube before the cutting process. Then, the processed straw is wrapped with three layers of medical PU film. This film has excellent adhesiveness and adheres well to the straws, ensuring airtightness of the actuator. The processed silicone heat-shrink tube is then fitted over the straws, ensuring that the incision positions align. A heat gun is used to shrink the tube so it fits tightly over the straw. Holes for securing the elastic fibers are created at both ends of the processed straw layer using a soldering iron. The elastic fiber is fibered through these holes and fixed at one end. It is pre-stretched to induce initial bending in the actuator before being secured at the other end. To ensure airtightness, silicone adhesive is applied to seal the holes. The soft actuator has an outer diameter of only 6 mm and a weight of just 1.9 g.


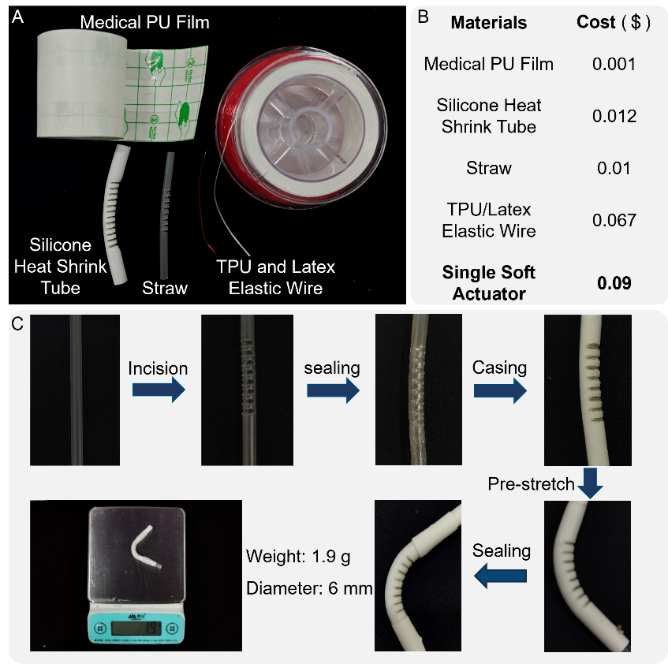


**Figure S1. Materials and Fabrication Process of the Soft Actuator.** A. Materials used for the fabrication of the soft actuator. B. The production cost of a single soft actuator. C. Fabrication process of the soft actuator.

The "force-strain" test experiment involved six groups of elastic fibers, including 1.0 mm TPU fibers, latex fibers, and non-elastic fibers, as shown in Figure S3(A). Each group varied in fiber type or length. The type of fiber affected the intrinsic mechanical properties, while variations in length led to different deformations during the tensile test, thus altering the "force-strain" constitutive relationship of each set. The tensile testing setup is illustrated in Figure S3(B), where the elastic fibers were uniformly stretched at a speed of 2 mm/s by a force gauge (DS2-5N, supplied by Zhique Precision Instruments Co., Ltd.) fixed on the moving platform, with the resulting "force-strain" data transmitted to a computer in real-time.


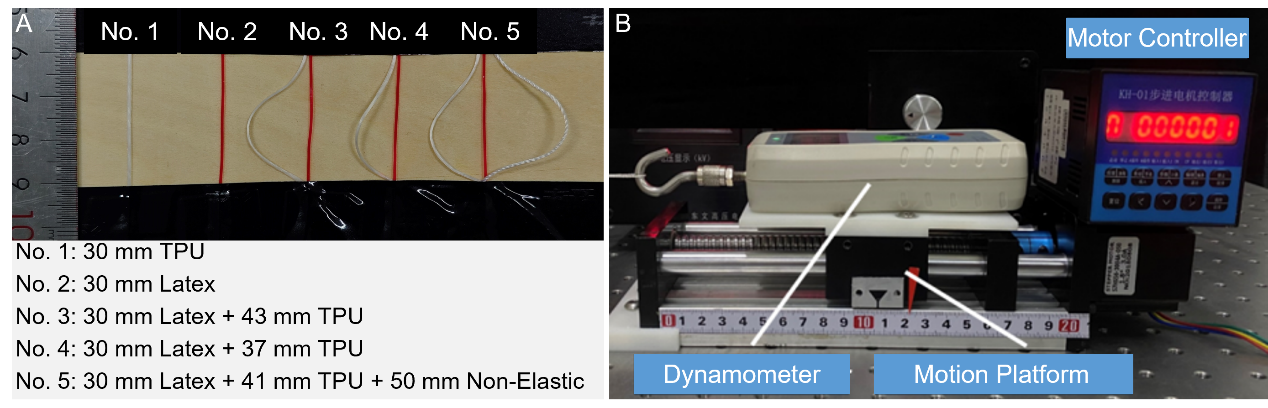


**Figure S2.** **"Force-Strain" Test Experiments for Different Elastic Fiber Sets.** A. Elastic fiber groups with different materials and initial lengths. B. Tensile testing platform.

Since the silicone heat-shrink tube used in this study lacks standard parameters, uniaxial tensile tests were conducted to measure the stress-strain data of the material, allowing for more accurate modeling of the soft actuator's hyperelastic properties. As shown in Figure S4(A), an electronic tensile testing machine (Zwick-010 1KN, ZwickRoell) was used, following the GB/T 528-2009 standard for uniaxial tensile performance tests on silicone heat-shrink tube specimens. The loading speed was set to 4 mm/min, and the resulting stress-strain curve is shown in Figure S4(B).


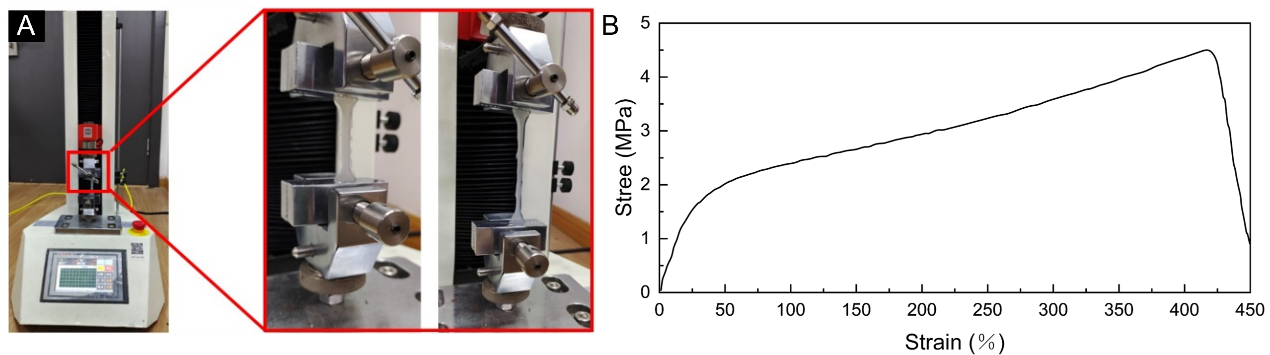


**Figure S3. Mechanical Properties Testing of Silicone Tubing.** A. Tensile testing setup; B. Stress-strain data chart.

**Theoretical model of pneumatic response characteristics of programmable soft actuator.**

This study achieves motion programming through the design of the elastic constraints of the soft actuator. The underlying principle relies on the distinct linear "force-deformation" response characteristics of elastic fibers with varying materials and diameters. By combining multiple elastic fibers, individual linear responses are synthesized into a predefined nonlinear mechanical property, thereby programming the actuator's response to input pressure into a specific nonlinear deformation output.

The "force-deformation" response curve of the elastic fiber combination can be expressed mathematically. Assuming that the combination consists of $n$ elastic fibers, the force function corresponding to the pre-stretching of each elastic fiber is given by:

$$\begin{aligned} F_{i}\left( x \right)=k_{i}x_{i}\#\left( 1 \right) \end{aligned}$$

where $k_{i}$ represents the elastic coefficient of the $i$-th elastic fiber, and $x_{i}$ denotes the deformation of the $i$-th fiber.

$$\begin{aligned} F\left( x \right)=F_{1}\left( x \right)+F_{2}\left( x \right)+\ldots+F_{n}\left( x \right)=\sum_{i=1}^{n} {(k}_{i}x_{i})\#\left( 2 \right) \end{aligned}$$

To simplify the calculation, we substitute $x_{1}$ and the deformation difference $\Delta x_{i}$ in place of $x_{i}$ in Equation (2). Let the deformation difference between the second elastic fiber and the first elastic fiber be $\Delta x_{1}$, and similarly, the deformation difference between the $n$-th elastic fiber and the $(n-1)$-th elastic fiber be $\Delta x_{n-1}$. Thus, the force equation for the $i$-th elastic fiber can be expressed as:

$$\begin{aligned} F_{i}\left( x \right)=k_{i}\left( x_{1}+\Delta x_{i-1}+\Delta x_{i-2}+\cdot\cdot\cdot+\Delta x_{1} \right)\#\left( 3 \right) \end{aligned}$$

Thus, the total force of the elastic fiber assembly can be expressed as:

$$\begin{aligned} F\left( x \right)=\sum_{i=1}^{n} k_{i}x_{1}+LA\#\left( 4 \right) \end{aligned}$$

Where $L=\left[ \begin{matrix} \Delta x_{1} & 0 & \ldots& 0 \\ \Delta x_{1} & \Delta x_{2} & \ldots& 0 \\ \vdots& \vdots& \vdots& 0 \\ \Delta x_{1} & \Delta x_{2} & \ldots& \Delta x_{n-1} \end{matrix} \right]$, $A=\left( \begin{matrix} k_{2} & k_{3} & \cdots& k_{n} \end{matrix} \right)$.

By adjusting the type, quantity, and deformation differences of the elastic fibers, a range of "force-deformation" response characteristics can be realized within the elastic fiber assembly. This, in turn, allows the soft actuator to exhibit distinct pneumatic response characteristics, thereby facilitating the motion programming of the actuator.

The silicone rubber used in this study exhibits an elongation of up to 400% and can be characterized by defining its strain energy density function. In the theoretical models of strain energy density, the most common include the Mooney-Rivlin model and the Yeoh model. The Mooney-Rivlin model is suitable for simulating moderate deformations with maximum tensile strain under 25% and pure shear strain under 75%, whereas the Yeoh model is more appropriate for large deformation scenarios. Given that the silicone rubber material used in this study is hyper-elastic and capable of large deformations, the Yeoh model was deemed more suitable.

$$\begin{aligned} W=W\left( I_{1}，I_{2}，I_{3} \right)\#\left( 5 \right) \end{aligned}$$

Where in equation (5):

$$\begin{aligned} \begin{aligned} &I_{1}=\lambda_{1}^{2}+\lambda_{2}^{2}+\lambda_{3}^{2} \\ &I_{2}=\lambda_{1}^{2}\lambda_{2}^{2}+\lambda_{2}^{2}\lambda_{3}^{2}+\lambda_{1}^{2}\lambda_{3}^{2} \\ &I_{3}=\lambda_{1}^{2}\lambda_{2}^{2}\lambda_{3}^{2} \\ &\lambda_{i}=1+\gamma_{i} \end{aligned}\#\left( 6 \right) \end{aligned}$$

Here, $I_{1}$, $I_{2}$, and $I_{3}$ are the tensor invariants of the Cauchy-Green strain, while $\lambda_{1}$, $\lambda_{2}$, and $\lambda_{3}$ represent the principal stretch ratios in the axial, circumferential, and radial directions of the actuator, respectively. $\gamma_{i}$ denotes the strain in the principal axial direction.

Combining the strain energy density function of the two-parameter Yeoh model with the above equations, it can be obtained as follows:

$$\begin{aligned} W=C_{10}\left( I_{1}-3 \right)+C_{20}\left( I_{1}-3 \right)^{2}\#\left( 7 \right) \end{aligned}$$

$$\begin{aligned} \frac{t_{1}}{2\left( \lambda_{1}-\frac{1}{\lambda_{1}^{2}} \right)}=C_{10}+\frac{1}{\lambda_{1}}C_{20}\#\left( 8 \right) \end{aligned}$$

Since the shell is made of silicone rubber, which is an incompressible material, the Cauchy-Green strain tensors exhibit the following property:

$$\begin{aligned} \lambda_{2}^{2}=\lambda_{3}^{2}=\frac{1}{\lambda_{1}}\#\left( 9 \right) \end{aligned}$$

Then we can get:

$$\begin{aligned} \left\{ \begin{aligned} I_{1}=\lambda_{1}^{2}+\frac{2}{\lambda_{1}} \\ I_{2}=2\lambda_{1}+\frac{1}{\lambda_{1}^{2}} \\ I_{3}=\left( \lambda_{1}\lambda_{2}\lambda_{3} \right)^{2}=1 \end{aligned} \right.\#\left( 10 \right) \end{aligned}$$

The stress-strain relationship for hyper-elastic materials can be derived based on its definition:

$$\begin{aligned} t_{ij}=\frac{\partial w}{\partial\lambda_{ij}}=\frac{\partial w\partial I_{1}}{\partial I_{1}\partial\lambda_{ij}}+\frac{\partial w\partial I_{2}}{\partial I_{2}\partial\lambda_{ij}}+\frac{\partial w\partial I_{3}}{\partial I_{3}\partial\lambda_{ij}}\#\left( 11 \right) \end{aligned}$$

The relationship between the principal axial force $t_{1}$ and the principal stretch ratio $\lambda_{1}$ is obtained as follows:

$$\begin{aligned} t_{1}=\frac{2}{\lambda_{1}}\left[ \frac{\partial w}{\partial I_{1}}+\left( \lambda_{2}^{2}+\lambda_{3}^{2} \right)\frac{\partial w}{\partial I_{2}}+\left( \lambda_{2}^{2}\lambda_{3}^{2} \right)\frac{\partial w}{\partial I_{3}} \right]\#\left( 12 \right) \end{aligned}$$

By combining equations (6) and (12) and deriving, we get:

$$\begin{aligned} \frac{t_{1}}{2\left( \begin{matrix} \lambda_{1} & -\frac{1}{\lambda_{1}^{2}} \end{matrix} \right)}=C_{10}+\left[ 2\left( \lambda_{1}^{2}+\frac{2}{\lambda_{1}} \right)-6 \right]C_{20}\#\left( 13 \right) \end{aligned}$$

The material parameters ($C_{10}$, $C_{20}$) in the two-term strain energy density function of the Yeoh model can be obtained through experiments on the mechanical properties of the material. By fitting the stress-strain data from uniaxial tensile tests of the silicone rubber, the parameters were determined to be $C_{10}=$0.381 and $C_{20}=$0.106.

The following assumptions must be satisfied for the static modeling: (1) The hyper-elastic material is incompressible, meeting the requirements of the Yeoh model. (2) The wall thickness of the actuator does not change during deformation. (3) The effect of the actuator's self-weight is negligible. (4) The elastic material is isotropic during deformation. By integrating the strain energy density function $W$ over the volume $V$ of the actuator, the elastic potential energy $U_{R}$ generated by the compression of the soft actuator can be obtained:

$$\begin{aligned} U_{R}=\int_{0}^{V} WdV\#\left( 14 \right) \end{aligned}$$

Thus, the theoretical energy equation for the soft actuator can be expressed as:

$$\begin{aligned} U_{A}=U_{R}+U_{L}=\int_{0}^{V} WdV+\frac{1}{2}\sum_{i=1}^{n} k_{i}{x_{i}}^{2}\#\left( 15 \right) \end{aligned}$$

Assume that during the expansion process of the hyper-elastic material, there is no conversion of internal energy, implying no change in heat. Due to the viscoelastic properties of hyper-elastic materials, deformation occurs at a very slow rate under load. In the absence of any external forces, when the inflation process within the pneumatic soft actuator's cavity is complete, the system reaches a steady state, and no kinetic energy change occurs during deformation. Thus, the work done by the gas pressure on the soft actuator is converted into the strain energy of the actuator and the elastic potential energy of the elastic filaments. According to the law of energy conservation, we have:

$$\begin{aligned} P_{i}dV_{c}{+WdV}_{r}=\sum_{i=1}^{n} k_{i}x_{i}dx_{i}\#\left( 16 \right) \end{aligned}$$

Where $P_{i}$ represents the air pressure input into the chamber of the soft actuator, $V_{c}$ and $V_{r}$ represents the chamber volume and the material volume of the soft actuator when the bending angle is $\theta$, respectively.

$$\begin{aligned} V_{c}=\pi\left( \frac{d}{2} \right)^{2}\left[ L_{0}+\Delta l-\left( \frac{d}{2}+t \right)\theta\right]\#\left( 17 \right) \end{aligned}$$

$$\begin{aligned} V_{r}=\pi\left[ \left( \frac{d}{2}+t \right)^{2}-\left( \frac{d}{2} \right)^{2} \right]\cdot\left[ L_{0}+\Delta l-\left( \frac{d}{2}+t \right)\theta\right]\#\left( 18 \right) \end{aligned}$$

From Equation (3), the deformation length of the $i$-th elastic fiber can be expressed in terms of the deformation length of the first elastic fiber as:

$$\begin{aligned} x_{i}=x_{1}+\Delta x_{i-1}+\Delta x_{i-2}+\cdot\cdot\cdot+\Delta x_{1}\#\left( 19 \right) \end{aligned}$$

From the geometric relationship, we obtain:

$$\begin{aligned} x_{1}=\Delta l-\left( d+t \right)\theta\#\left( 20 \right) \end{aligned}$$

Through derivation, the relationship between the inflation pressure $P$ and the bending angle $\theta$ can be expressed as:

$$\begin{aligned} P_{i}=\frac{8\sum_{i=1}^{n} k_{i}(d+t)x_{i}-8\pi\left( dt+t^{2} \right)\left[ L_{0}+\Delta l-(\frac{d}{2}+t)\theta\right]\frac{dw}{d\theta}}{\pi d^{2}\left( d+2t \right)}\#\left( 21 \right) \end{aligned}$$

The soft actuator has a diameter of 6 mm, and the elastic fiber used is a 1 mm diameter TPU fiber. The pressure value $P$ ranges within 300 kPa, starting from 3 kPa and incrementing in 3 kPa intervals. Using Origin, the corresponding bending angles of soft actuators with initial bending angles of 90°, 105°, and 120° were calculated.

Based on the theoretical formulations above, we can also determine the initial bending angle of the actuator by selecting the material and pre-stretch ratio of the elastic fibers. At this point, with an input pressure $P$ of 0 kPa and the incision design parameters already optimized and determined, the initial bending angle depends solely on the elastic fiber material and its pre-stretch length. TPU elastic fibers with diameters of 0.6 mm and 1.5 mm, as well as a 1 mm latex fiber, were employed in fabricating the actuator. By adjusting the pre-stretch length, the initial bending angle of the actuator can be predicted. As shown in Table S1, different elastic fiber combinations and their pre-stretch lengths are associated with the theoretical and experimental values of the initial bending angle, as presented in Figure S1(A). Using the theoretical formulations, we derived the relationship between the elastic fiber material, pre-stretch length, and the initial bending angle, as shown in Figure S1(B). This relationship enables greater flexibility in designing the initial bending angle by allowing a broader selection of elastic fiber materials and pre-stretch lengths. For the same initial bending angle, soft actuators constrained by different elastic fiber materials exhibit different actuation response characteristics, as shown in Figure S1(C). Therefore, by selecting appropriate elastic fiber materials and pre-stretch lengths, the initial bending angle and pneumatic response characteristics of the actuator can be determined.

Table S1. Several Elastic Fiber Combinations

| Elastic fiber groups | Elastic fiber arrangement |
| --- | --- |
| Group 1 | 0.6 mm diameter TPU elastic fiber |
| Group 2 | 1.5 mm diameter TPU elastic fiber |
| Group 3 | 1 mm diameter latex elastic fiber |
| Group 4 | 0.6 mm diameter TPU + 1 mm diameter latex elastic fiber |
| Group 5 | 1.5 mm diameter TPU + 1 mm diameter latex elastic fiber |


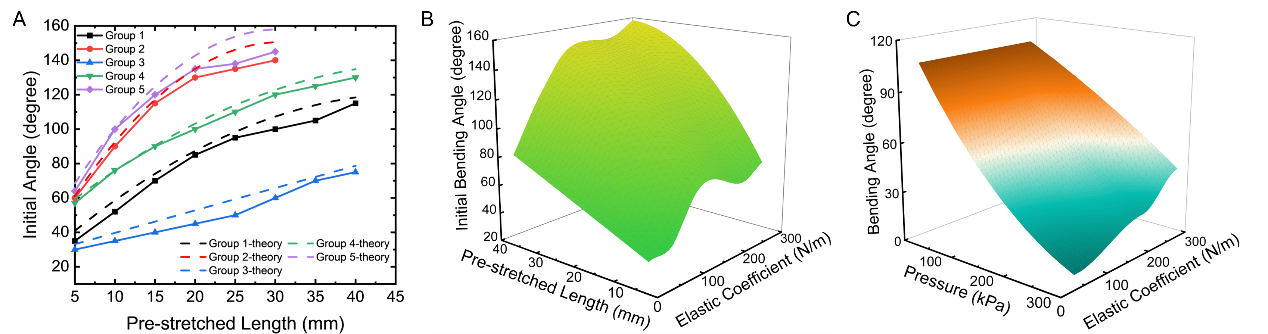


**Figure S4. Theoretical Predicted Effects of Elastic Fiber Arrangement and Input Pressure on Bending Soft Actuators.** A. Theoretical analysis and experimental comparison of the effect of different elastic fiber materials and pre-stretch lengths on the initial bending angle of the actuator. B. 3-D plot of the theoretically predicted initial bending angle as a function of pre-stretched length and elastic coefficient of elastic fibers. C. 3-D plot of the theoretically predicted bending angle as a function of input pressure and elastic coefficient of elastic fibers.

The pneumatic actuation control experimental platform was established for performance testing and application experiments, as shown in Figure S5(A). The experimental platform is composed of three main parts: the pneumatic circuit, control circuit, and data transmission. The pneumatic circuit supplies the required pressure for testing; the control circuit handles all commands during testing, including output frequency and pressure control signals; data transmission refers to processing the experimental data via a computer. Overview of the pneumatic actuation control experimental platform is shown in Figure S5(B).


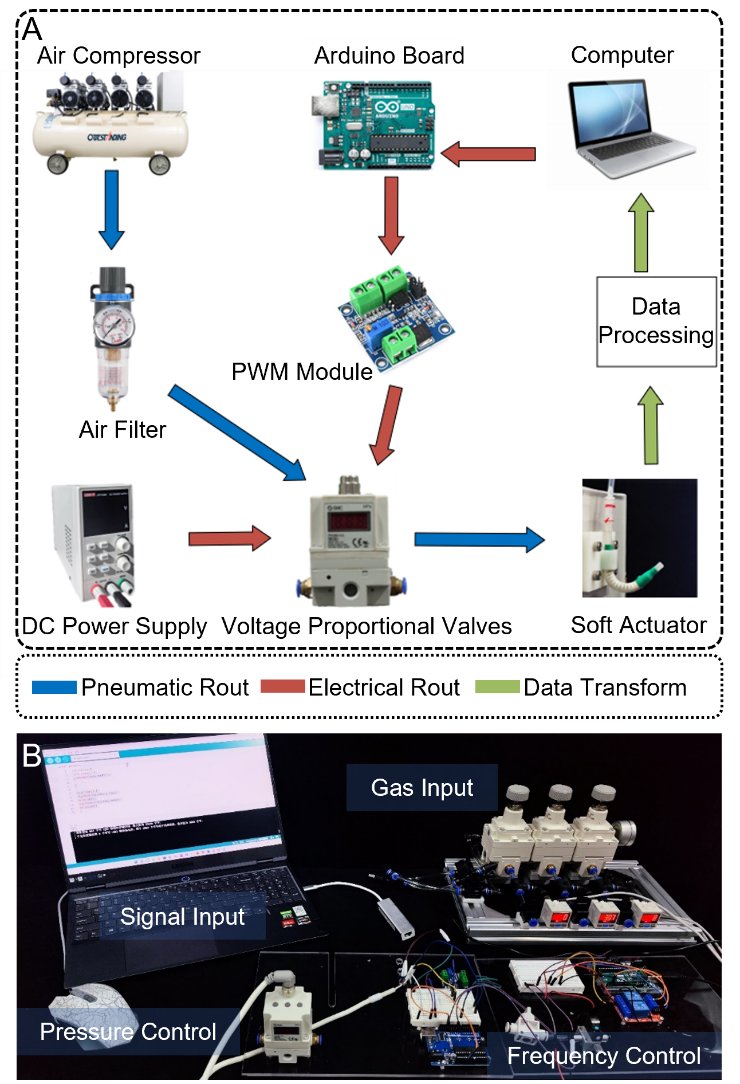


**Figure S5. Design of the Pneumatic-Driven Control Experimental Platform.** A. Schematic diagram of the experimental platform; B. Pneumatic-driven control experimental platform.

The soft actuator output torque testing platform is shown in Figure S6. The force sensor (DS2-5N, Zhique Precision Instruments Co., Ltd.) was positioned at the tip of the soft actuator. The pneumatic pressure of the actuator was controlled by an electronic pressure regulator (ITV1030-312N, SMC). The input driving pressure begins at 30 kPa, incremented in 30 kPa steps up to 300 kPa. Experimental results of the input pressure and output torque of the soft actuator at different initial bending angles are presented in Figure 4D.


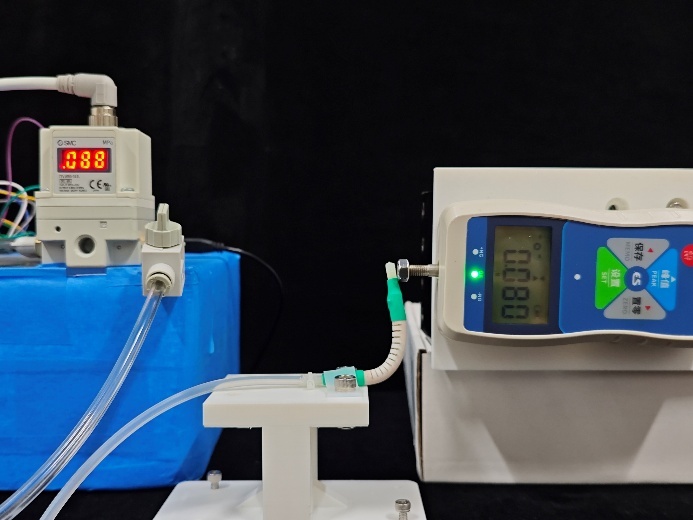


**Figure S6. Actuator Output Force Performance Testing Experiments.**

The omnidirectional crawling soft robot consists of Modules A, B, and C, where Modules B and C collectively determine the robot’s movement direction. The bending angle variations of Modules B and C across different pressure ranges are depicted in Figure S7. When the input pressure ranges from 0 to 120 kPa, Module B exhibits a motion range of 70° to 105°, whereas Module C exhibits a range of 80° to 105°. Given that the bending angle variation of Module B exceeds that of Module C under these conditions, the resultant motion induces a rightward turning movement of the soft robot. When the input pressure increases to 200 to 300 kPa, Module B maintains a motion range of 70°, whereas Module C exhibits a range of 50° to 70°. As the bending angle variation of Module B is smaller than that of Module C, the resultant motion induces a leftward turning movement.


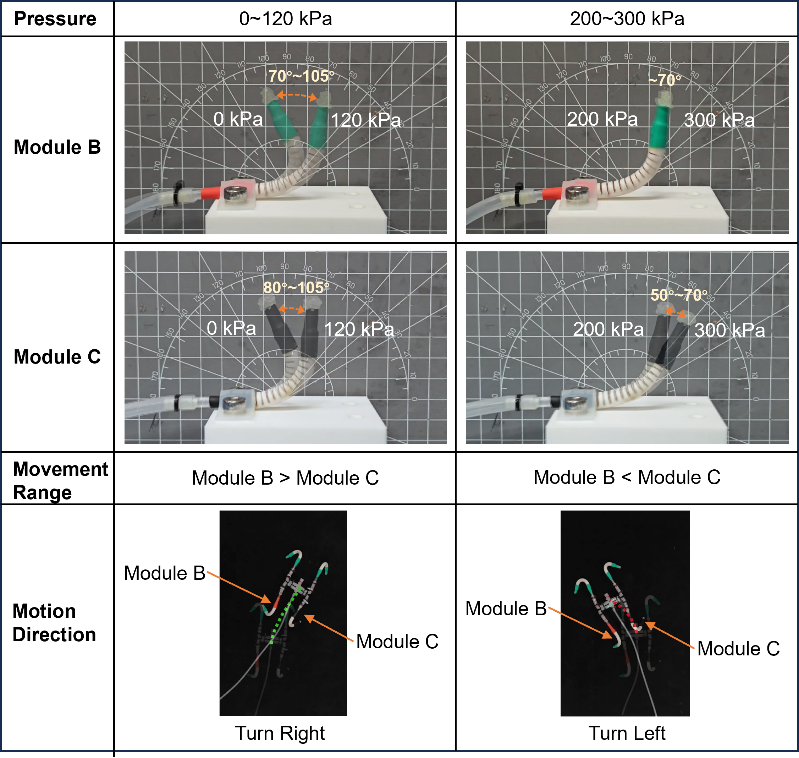


**Figure S7. Variations in the bending angles of Modules B and C under different pressure ranges.**
